# Coxsackievirus B3 Exploits the Ubiquitin-Proteasome System to Facilitate Viral Replication

**DOI:** 10.3390/v13071360

**Published:** 2021-07-13

**Authors:** Martin Voss, Vera Braun, Clara Bredow, Peter-Michael Kloetzel, Antje Beling

**Affiliations:** 1 Institute of Biochemistry, Charité-Universitätsmedizin Berlin, Corporate Member of Freie Universität Berlin and Humboldt-Universität zu Berlin, 14195 Berlin, Germany; Martin.Voss81@gmx.net (M.V.); vera.braun2@charite.de (V.B.); clara.bredow@charite.de (C.B.); p-m.kloetzel@charite.de (P.-M.K.); 2 Deutsches Zentrum für Herz-Kreislauf-Forschung, Partner Site Berlin, 10785 Berlin, Germany

**Keywords:** ubiquitin-proteasome system, virus, infection, coxsackievirus, proteasome inhibition

## Abstract

Infection by RNA viruses causes extensive cellular reorganization, including hijacking of membranes to create membranous structures termed replication organelles, which support viral RNA synthesis and virion assembly. In this study, we show that infection with coxsackievirus B3 entails a profound impairment of the protein homeostasis at virus-utilized membranes, reflected by an accumulation of ubiquitinylated proteins, including K48-linked polyubiquitin conjugates, known to direct proteins to proteasomal degradation. The enrichment of membrane-bound ubiquitin conjugates is attributed to the presence of the non-structural viral proteins 2B and 3A, which are known to perturb membrane integrity and can cause an extensive rearrangement of cellular membranes. The locally increased abundance of ubiquitinylated proteins occurs without an increase of oxidatively damaged proteins. During the exponential phase of replication, the oxidative damage of membrane proteins is even diminished, an effect we attribute to the recruitment of glutathione, which is known to be required for the formation of infectious virus particles. Furthermore, we show that the proteasome contributes to the processing of viral precursor proteins. Taken together, we demonstrate how an infection with coxsackievirus B3 affects the cellular protein and redox homeostasis locally at the site of viral replication and virus assembly.

## 1. Introduction

Coxsackievirus B3 (CVB3) is a positive-sense single stranded RNA virus that belongs to the genus Enterovirus of the family Picornaviridae. CVB3 is widely distributed and a common pathogen in human. Although most infections with CVB3 are subclinical or cause only flu-like symptoms, in infants and physically active individuals CVB3 can trigger severe conditions like aseptic meningitis or viral myocarditis that may promote dilated cardiomyopathy and cardiac failure [1]. The non-enveloped virion has an icosahedral symmetry with a diameter of approximately 30 nm, enters target cells via receptor-mediated endocytosis and releases a 7.4 kb RNA genome into the cytoplasm [2]. The viral genome is translated into a single polyprotein that is co-translationally subjected to a series of autocleavages, generating individual proteins. The eventually released viral proteins include four capsid proteins (VP1–VP4) and seven non-structural proteins (2A–C, 3A–D) as well as some stable and functional precursor proteins (2BC, 3AB, 3CD). The initial cleavage of the polyprotein is catalyzed by the viral protease 2A, which releases the fragment P1 that contains the capsid proteins. The further cleavage of P1 and the processing of the fragments P2 (2A–C) and P3 (3A–D) are catalyzed by the protease 3C and its stable precursor 3CD.

The non-structural proteins of all positive strand RNA viruses are involved in viral RNA replication but also interfere and even exploit normal cellular functions, including protein degradation pathways [3]. In eukaryotic cells, the ubiquitin-proteasome system (UPS) serves as the major pathway for extralysosomal protein degradation and involves two successive steps: [1] ubiquitinylation (also referred to as ubiquitylation or ubiquitination), which involves the covalent attachment of ubiquitin chains to the target protein substrate, and [2] proteasome-mediated proteolysis of the ubiquitinylated protein and release of re-usable ubiquitin [4]. Ubiquitin is a 76-amino-acid protein that is highly conserved from yeast to man. In poly-ubiquitin chains, eight types of linkages have been identified, which are formed by the covalent attachment of the C-terminus of ubiquitin to any of the seven lysine residues (K6, K11, K27, K29, K33, K48, K63) or the N-terminal methionine residue (M1) in the preceding ubiquitin [5]. K48-linked polyubiquitin is the most abundant linkage type and the canonical signal for degradation of the tagged substrate protein by the proteasome. The function of other linkage types may extend beyond proteasomal degradation. K63-linked polyubiquitin, for example, is involved in several signaling pathways.

Protein ubiquitinylation constitutes a reversible protein modification that extends beyond the proteasome-mediated degradation of proteins and is involved in the regulation of many cellular processes [6]. In this study, we characterize the alterations in UPS-dependent protein homeostasis during infection with CVB3 and demonstrate that the activity of the proteasome is exploited for the processing of the viral precursor proteins.

## 2. Materials and Methods

### 2.1. Cell Culture and Transient Transfection

HeLa cells (ATCC, Manassas, VA, USA) were maintained in DMEM supplemented with 10% (*v*/*v*) fetal bovine serum and 1% (*v*/*v*) penicillin/streptomycin. For transient transfections, HeLa cells were grown to 90% confluence and transfected with 0.5 µg expression vector per 1 × 10^5^ cells using Polyethylenimine–Linear, MW 25000 (Polyscience, Inc., Hirschberg Germany). Synthetic genes of CVB3 (strain Nancy) proteins were generated by Integrated DNA Technologies, Inc (Coralville, IA, USA), and cloned into pFN21A-HaloTag Flexi (Promega, Fitchburg, WI, USA), or pEGFP-C3 (Clontech, Mountain View, CA, USA) expression vector. The medium was replaced 6 h after transfection.

### 2.2. Virus Stocks

HeLa cells were infected with coxsackievirus B3 strain Nancy (K. Klingel, University of Tübingen) at indicated multiplicity of infection (MOI). Virus titers were determined by plaque assay and aliquots stored at −80 °C. Plaque assays were performed on sub-confluent monolayers of HeLa cells incubated with serial 10-fold dilutions of cell culture supernatant. After incubation at 37 °C for 30 min, supernatants were removed, and monolayers were overlaid with agar containing Eagle’s minimal essential medium (MEM) and 10% FCS. After 2 days, virus plaques were stained with 0.5% MTT/PBS (3-(4,5-dimethylthiazol-2-yl)-2,5-diphenyltetrazolium bromide; Sigma, Saint Louis, MO, USA).

### 2.3. Assay of Cell Viability

For the analysis of cell viability by flow cytometry, cells were collected in PBS supplemented with 2% FBS and 2 mM EDTA, and incubated on ice with LIVE/DEAD™ Fixable Far Red stain (Thermo Scientific, Seattle, MA, USA ) for 30 min. Subsequently, the cell pellet was resuspended in Roti-Histofix (Carl Roth, Karlsruhe, Germany) and incubated at room temperature for 15 min. Excess formaldehyde was quenched by adding a four-fold volume of PBS supplemented with 2% FBS and 2 mM EDTA. Samples were measured using the platform FACSCalibur (BD Biosciences, Heidelberg, Germany) and analyzed with the software FlowJo 10.6.1 (FlowJo, Ashland, OR, USA).

### 2.4. Protein Extraction and Anti-HA Pull-Down

Adherent cultures of HeLa cells were lysed in 20 mM Hepes pH 7.4, 0.5% (*v*/*v*) CHAPS, 8 mM EDTA, 2 mM EGTA, complete protease inhibitor (Roche, Basel, Switzerland), 50 mM NaF, 5 mM Na-pyrophosphate, 2 mM Na-o-vanadate, 10 mM NEM. Cell lysates were incubated on ice for 20 min, and then centrifuged at 16,000 rcf (4 °C) for 10 min to pellet debris. Protein concentration was determined by Bradford assay.

For immunoprecipitation of HA tagged proteins, cleared lysates were incubated at 4 °C with anti-HA magnetic beads (Thermo Scientific, Seattle, MA, USA) for 60 min. The magnetic beads were washed three times with lysis buffer, followed by a wash with 300 mM NaCl in 20 mM Hepes pH 7.4, and a final wash with 0.1 mM EGTA in 20 mM Hepes pH 7.4. Bound protein was eluted with SDS-PAGE sample buffer.

### 2.5. Differential Centrifugation

Adherent cultures of HeLa cells were scraped off, pelleted by centrifugation at 200 rcf for 3 min, washed in PBS, pelleted again, and resuspended in 20 mM HEPES pH 7.4, 10 mM KCl, 2 mM MgCl_2_, 1 mM EDTA, 1 mM EGTA, complete protease inhibitor (Roche, Basel, Switzerland), 10 mM NEM. Cell suspension was incubated on ice for 10 min, and then passed 10 times through a 30-gauge needle. Intact cells were pelleted by centrifugation at 200 rcf for 5 min (4 °C) and resulting supernatant transferred to a new tube for Bradford assay and subsequent differential centrifugation. Crude lysate with 2 µg/µL protein was centrifuged at 16,000 rcf for 10 min (4 °C), supernatant transferred to polycarbonate tubes and centrifuged at 120,000 rcf (Beckman Coulter Optima TLX, Beckman Coulter, Brea, CA, USA) for 60 min (4 °C). The supernatant and membrane pellets were immediately used for further analysis.

### 2.6. Immunoblotting

SDS-PAGE was performed on 12%, 15%, or 4–15% (Bio-Rad) Tris-glycine gels using Tris-glycine running buffer. For SDS-PAGE protein samples were prepared in 62.5 mM Tris HCl pH 6.8, 10% Glycerol, 2% SDS, 0.005% Bromophenol Blue. The semi-dry transfer (Bio-Rad) of proteins onto 0.22-µm nitrocellulose membrane (LI-COR Biosciences, Lincoln, NE, USA) was carried out discontinuous Tris-CAPS buffer. Immunostaining was performed according to standard protocols. The following primary antibodies were used: HA (Abcam, Cambridge, UK), GAPDH (Thermo Scientific, Seattle, MA, USA), VP1 (Mediagnost, Reutlingen, Germany), VP1 (Dako, Hamburg, Germany), LC3-A/B (Cell Signaling, Danvers, MA, USA), p62 (Enzo, Farmingdale, NY, USA), ERp72 (Cell Signaling, Danvers, MA, USA), RCAS1 (Cell Signaling, Danvers, MA, USA), mono-/poly-ubiquitin (Enzo, Farmingdale, NY, USA), K48-linked ubiquitin (Cell Signaling, Danvers, MA, USA), K63-linked ubiquitin (Enzo, Farmingdale, NY, USA), ribosomal protein S3 (Abcam, Cambridge, UK), ribosomal protein S6 (Abcam, Cambridge, UK), proteasomal subunit beta 2 (Abcam, Cambridge, UK), proteasomal subunit beta 5 (Abcam, Cambridge, UK), Halo (Promega, Fitchburg, WI, USA), and DNP (Sigma-Aldrich, Sant Louis, MO, USA). Antibodies recognizing CVB3 proteins 2C, 3D, and VP423 were provided by K. Klingel (University of Tübingen, Germany). Secondary IRD680CW or IRDye800CW labeled antibodies (LI-COR Biosciences, Lincoln, NE, USA) were visualized using an Odyssey CLx imager and analyzed with Image Studio software 5.2 (LI-COR Biosciences, Lincoln, NE, USA).

### 2.7. Ellman’s Test

For the detection of free SH groups, samples were supplemented with 200 µM of 5,5′-dithiobis-(2-nitrobenzoic acid) (Sigma-Aldrich, Saint Louis, MO, USA) in 100 mM Tris pH 8, incubated for 20 min, and absorbance measured at 412 nm in a Genesys 20 spectrophotometer (Thermo, Seattle, MA, USA). Cleared total lysates (2 µg/µL) or cytosolic fractions, obtained by ultracentrifugation from cleared lysates (2 µg/µL), were assayed in 1:10 dilutions. Membrane fractions were directly resuspended in assay buffer with a volume corresponding to the cytosolic fraction. The SH concentration was calculated using a molar extinction coefficient of 14,150 M^−1^ cm^−1^.

### 2.8. OxyBlot

For detection of proteins modified with carbonyl groups, samples were incubated with 10 mM 2,4-Dinitrophenylhydrazine (Sigma-Aldrich, MO, USA) prepared as 100 mM stock solution in 50% trifluoroacetic acid. The reaction was neutralized by adding 1/3 volume of 2 M Tris. Samples were subjected to SDS-PAGE and DNP-hydrazone derivatized carbonyl groups in protein side chains were detected by immunoblotting using an anti-DNP antibody (Sigma-Aldrich, Saint Louis, MO, USA).

## 3. Results

### 3.1. Evasion of Proteasomal Degradation at CVB3-Utilized Membranes

Previous reports described an increase in protein ubiquitinylation in CVB3 infected cells and that the application of proteasome inhibitors reduces viral replication, suggesting that the UPS is exploited by the virus [7,8].

**Figure 1:**
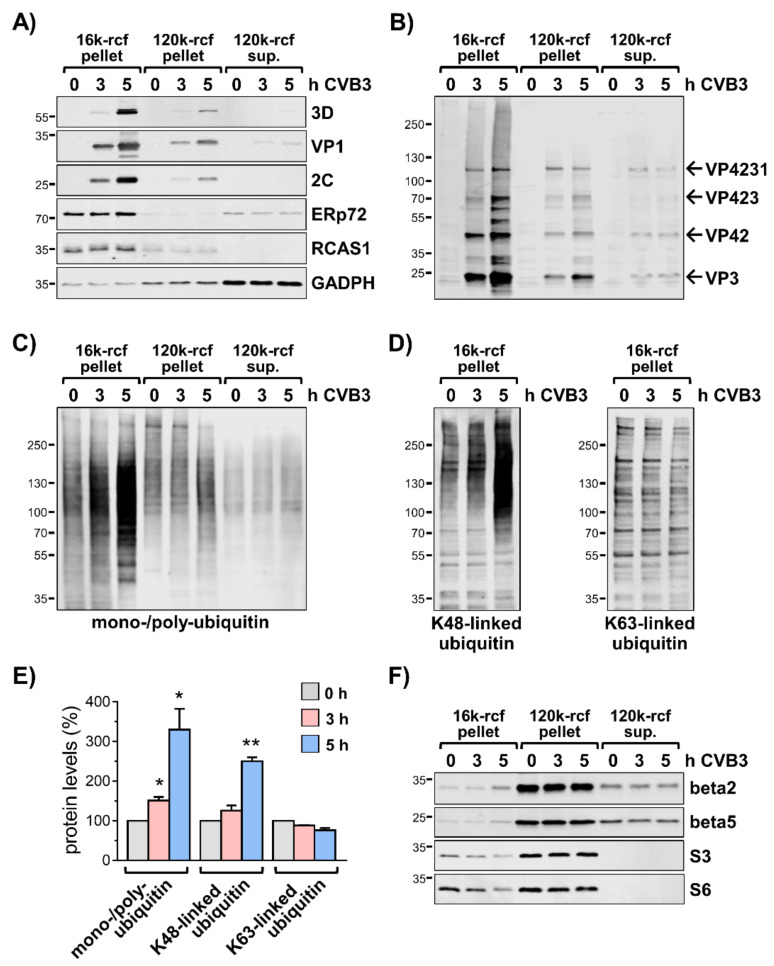
Accumulation of ubiquitin conjugates at CVB3-utilized compartments. (**A**) HeLa cells were treated for indicated periods of time −/+ CVB3 (MOI 1) and disrupted in hypotonic lysis buffer. Pre-cleared lysates were subjected to differential centrifugation at 16,000 rcf (16k) and 120,000 rcf (120k). Resulting membrane pellets and supernatants were analyzed by immunoblotting of CVB3 proteins 3D, VP1, and 2C, ER-marker ERp72, Golgi-marker RCAS1, and GAPDH. (**B**) Immunoblotting of fractionation samples using an antiserum raised against the fragment VP423 of the CVB3 polyprotein. The arrows indicate immunosignals of precursor proteins and cleavage products corresponding to VP4231 (~94 kDa), VP423 (~62 kDa), VP42 (~36 kDa), and VP3 (~26 kDa). (**C**) Immunoblotting of mono-/poly-ubiquitinylation in fractionation samples of CVB3 infected HeLa cells. (**D**) Immunoblots of K48-linked and K63-linked polyubiquitin in 16k-rcf membrane pellets. (**E**) The bar chart summarizes the densitometric analysis of anti-ubiquitin immunosignals in 16k-rcf fractions (0 h = 100%, *n* = 4). * *p* < 0.05, ** *p* < 0.01. (**F**) Immunoblotting of proteasomal subunits beta 2 and beta 5, and ribosomal subunits S3 and S6.

For a more detailed assessment of the effect of CVB3 infection on protein ubiquitinylation, extracts of HeLa cells were subjected to differential centrifugation to obtain a medium-speed membrane pellet (16k) with intact organelles and large vesicles, a high-speed membrane pellet (120k) containing microsomes as well as a high-speed supernatant representing the cytosolic fraction. Between three and five hours after start of infection, corresponding to the exponential phase of the first replication cycle, viral proteins were almost exclusively localized in the 16k membrane pellet as shown by immunoblotting of the non-structural protein 2C, the viral RNA-dependent RNA polymerase 3D, and the viral capsid protein VP1 (Figure 1A).

The predominant enrichment of viral proteins in the 16k membrane pellet indicates that this fraction contains the viral replication organelles (ROs), which constitute modified cellular membranes where viral replication complexes are concentrated and virion assembly occurs [9,10]. Indeed, the immunoblotting of the endoplasmic reticulum (ER) marker protein ERp72 and the Golgi marker RCAS1 showed that the 16k pellet contained membranes derived from ER and Golgi apparatus, which are targeted by the non-structural membrane proteins of CVB3 to generate ROs [11,12].

Immunoblotting of fractionation samples confirmed also the predominant localization of the polyprotein fragments VP4231, VP423, VP42, and VP3 in the 16k pellet, indicating that the actual polyprotein processing occurs at ROs, but also revealed a smear-like pattern that suggests poly-ubiquitinylation of these proteins (Figure 1B, Figure S1A). Thus, despite the location of viral proteins at membranes of ROs, they may still be targeted by the UPS. An anti-HA pull-down from lysates of HA-ubiquitin transfected cells infected with CVB3 confirmed the ubiquitinylation of viral capsid proteins (Figure S1B,C).

During the first replication cycle of CVB3 an overall accumulation of mono- and poly-ubiquitinylated proteins was observed in the 16k membrane fraction (Figure 1C). The enrichment of mono- and poly-ubiquitinylated proteins was much less pronounced in the 120k membrane pellet and the cytosolic fraction (Figure 1C). Of note, during the exponential phase of replication, the abundance of K48-linked poly-ubiquitin chains increased with progressing CVB3 replication (Figure 1D,E), indicating that proteins were tagged for proteasome-mediated proteolysis. In contrast, levels of K63-linked poly-ubiquitin with regulatory functions were not increased (Figure 1D,E).

We considered that the observed ubiquitin conjugates are attributed to co-translational ubiquitination of misfolded host or viral proteins directly synthesized at ROs. Indeed, the ribosomal subunits S3 and S6 were detected in 16k membrane fractions (Figure 1F), indicating the presence of ribosomes. However, the levels of S3 and S6 decreased with progressing CVB3 replication. The proteasome subunits beta2 and beta5 were mainly detected in the 120k membrane pellets and cytosolic fraction of both non-infected and infected cells with similar intensities. However, the signals of both proteasome subunits were also detectable in the 16k membrane pellets with slightly more pronounced intensity in samples collected five hours after start of infection (Figure 1F). Nevertheless, the subcellular distribution and intense accumulation of K48-linked ubiquitin conjugates shows that the process of ubiqutinylation is profoundly increased at CVB3-utilized membranes and/or that the modification of the membrane compartment might affect the proteasome-mediated degradation of associated proteins.

To preserve ubiquitin conjugates, the applied lysis buffer was supplemented with the alkylating agent N-ethylmaleimide (NEM) in order to inactivate ubiquitin peptidases [13]. Interestingly, when processing samples without NEM, the loss of ubiquitin conjugates during samples processing was significantly less pronounced in the 16k-rcf membrane pellet of CVB3 infected cells compared to non-infected cells (Figure S1D). Therefore, it is conceivable that the membrane modifications, which lead to the formation of ROs, might at least partially impair the deubiquitinylation or proteasomal degradation of ubiquitinylated proteins.

### 3.2. Accumulation of Ubiquitin Conjugates as a Result of Membrane Modifications

**Figure 2:**
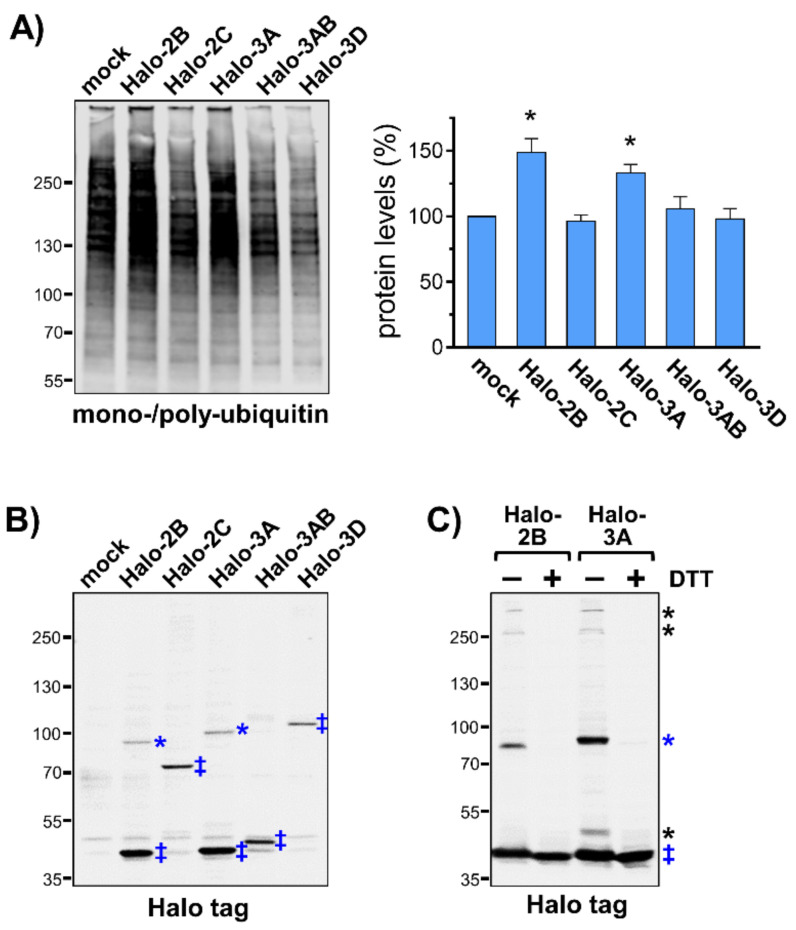
Accumulation of ubiquitin conjugates during ectopic expression of CVB3 proteins 2B and 3A. (**A**) HeLa cells transfected with indicated Halo-tagged CVB3 proteins for 48 h were collected in hypotonic buffer and subjected to centrifugation at 16,000 rcf (16k). The resulting 16k-rcf membrane pellets were analyzed by immunoblotting of mono-/poly-ubiquitinylation. The bar chart summarizes the densitometric analysis of anti-ubiquitin immunosignals in 16k-rcf fractions (mock = 100%, *n* = 3). * *p* < 0.05. (**B**) Immunoblotting of Halo tag in 16k-rcf membrane pellets of HeLa cells transfected with Halo-tagged CVB3 proteins: 2B (44 kDa), 2C (70.4 kDa), 3A (42.9 kDa), 3AB (45.3 kDa), and 3D (85.7 kDa). Double-cross indicates proteins at expected size, asterisks indicate SDS-resistant complexes corresponding to homodimers. (**C**) Halo-tagged CVB3 proteins 2B and 3A were expressed in HeLa cells and subjected to SDS-PAGE in sample buffer −/+ 100 mM DTT. Double-cross indicates proteins at expected size, blue asterisks indicate DTT-sensitive homodimers, black asterisks indicate DTT-sensitive heterodimeric complexes.

During CVB3 replication, ubiquitin conjugates are particularly enriched in the subcellular fraction that contained virus-utilized membrane compartments. This finding prompted us to investigate the effect of an ectopic expression of the non-structural CVB3 proteins 2B, 2C, 3A, 3AB, and 3D, which interact directly with cellular membranes via hydrophobic domains or binding to phospholipids [14,15]. The analysis of the 16k membrane fraction of HeLa cells transfected with Halo-tagged variants of these CVB3 proteins by immunoblotting showed an increase in ubiquitinylation in case of protein 2B as well as 3A compared to mock transfection or cells expressing the proteins 2C, 3AB, or 3D (Figure 2A). On the one hand, this finding demonstrates that the ectopic expression of the CVB3 proteins 2B or 3A is sufficient to cause an accumulation of ubiquitinylated membrane proteins. On the other hand, it argues against the above-mentioned possibility of co-translational ubiquitinylation of viral proteins during viral replication to be the main reason for the increased levels of ubiquitin conjugates during progressing viral replication. Moreover, the sequences of protein 2B and 3A include no signal for membrane targeting. Instead, the proteins insert their hydrophobic domains into cellular membranes after synthesis in the cytosol [16].

Interestingly, immunoblotting of the Halo-tagged viral proteins revealed that protein 2B and 3A are not only present as monomers but form also SDS-resistant complexes (Figure 2B). This was not observed for the viral proteins 2C, 3AB, and 3D. The preparation of protein samples in presence of the reducing agent dithiothreitol (DTT) resulted in a loss of the additional complexes, indicating that proteins 2B and 3A form most likely disulfide-linked dimers or even oligomers (Figure 2C). Importantly, the occurrence of disulfide-linked viral transmembrane proteins indicates oxidative modifications of membrane proteins.

The CVB3 proteins, 2B and 3A, are both involved in the disruption of the secretory pathway of infected cells and cause rearrangements of cellular membranes contributing to the formation of ROs [11,12]. Furthermore, protein 2B is known to form homotetramers, which act as viroporins and increase membrane permeability, e.g., for Ca^2+^ ions [17]. Protein 3A was previously also described as a viroporin, but it is not as well characterized as protein 2B in this regard [18]. A cell viability assay that was performed in this study strongly indicated that protein 3A of CVB3 increases also the permeability of membranes for Ca^2+^ ions (Figure S2). A disturbed ion homeostasis due to viroporin activity would most likely also affect the cellular redox homeostasis [19]. An increase in reactive oxygen species due to an impaired redox homeostasis might entail an increase in oxidatively damaged proteins that are known to be targeted by the UPS [20].

### 3.3. Modified Redox Homeostasis at Virus-Utilized Membranes

**Figure 3:**
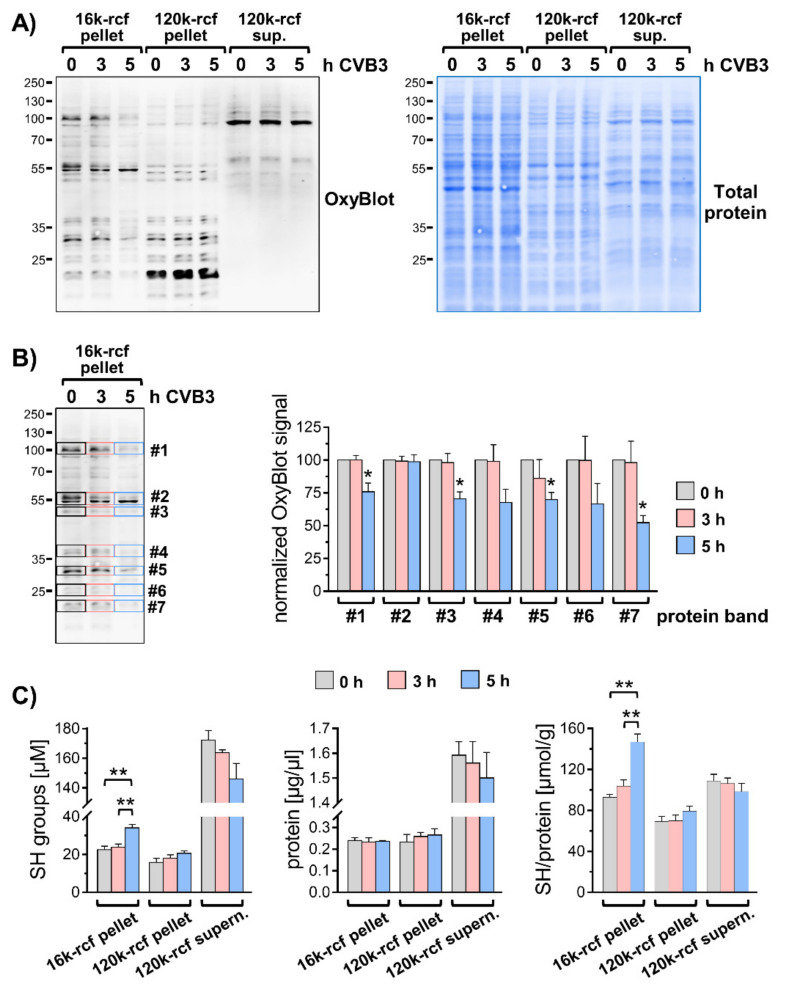
Analysis of oxidative modifications in subcellular fractions. (**A**–**C**) HeLa cells −/+ CVB3 (Nancy, MOI 1, 5 h) were disrupted in hypotonic lysis buffer using a 30-gauge syringe. Pre-cleared lysates (200 rcf for 5 min) were subjected to differential centrifugation at 16,000 rcf (16k) and 120,000 rcf (120k). (**A**) Resulting membrane pellets and supernatants were analyzed by OxyBlot and total protein staining. (**B**) Indicated bands #1–#7 of the 16k-rcf fractions were quantified (*n* = 4). The bar chart depicts the CVB3-dependent change of anti-DNP immunosignals normalized to the respective signal in non-infected sample (0 h = 100%), * *p* < 0.05. (**C**) Subcellular fractions were analyzed by Ellman’s test to determine concentration of free SH groups and subsequently by Bradford assay in order to normalize SH levels to respective protein concentrations (*n* = 4), ** *p* < 0.005.

To test the possibility that oxidatively damaged proteins contribute to the elevated levels of ubiquitin conjugates in CVB3 infected cells, fractionation samples were analyzed by OxyBlot, which allows detection of carbonyl groups introduced into proteins as a result of a reaction with free radicals or peroxides. Analysis by OxyBlot revealed that in untreated cells oxidatively modified proteins occur mainly in the 16k and 120k membrane fractions (Figure 3A). In the 120k pellet, and to a lesser extent in the 16k fraction, the oxidative modifications affected especially proteins < 40 kDa and were detected to a much lesser extent in the cytosolic fraction. This finding indicated that particularly membrane proteins are prone to oxidative modifications despite an overall normal redox homeostasis in non-challenged control cells. The degree of oxidative damage to membrane proteins in the 16k membrane pellet was reduced during the exponential phase of CVB3 replication (Figure 3A,B). This effect was not observed in the 120k membrane pellet or cytosolic fraction (Figure 3A). We verified that this was not attributed to altered protein levels by a total protein staining. The overall protein levels in the respective subcellular fractions were not profoundly altered in CVB3 infected cells as depicted (Figure 3A).

The phenomenon that the oxidative damage at virus-utilized membranes is reduced during the exponential phase of replication was attributed to the binding of redox-active glutathione by viral capsid proteins during virus assembly [21,22]. An assay of SH groups in fractionation samples by Ellman’s test confirmed for CVB3-infected cells the accumulation of small thiol compounds in the 16k membrane pellet (Figure 3C). Therefore, it can be excluded that the increase in ubiquitinylation in CVB3 infected cells is attributed to oxidative damage of membrane proteins.

### 3.4. The Effect of the Proteasome Inhibitor Epoxomicin Is Diminished during CVB3 Infection

**Figure 4:**
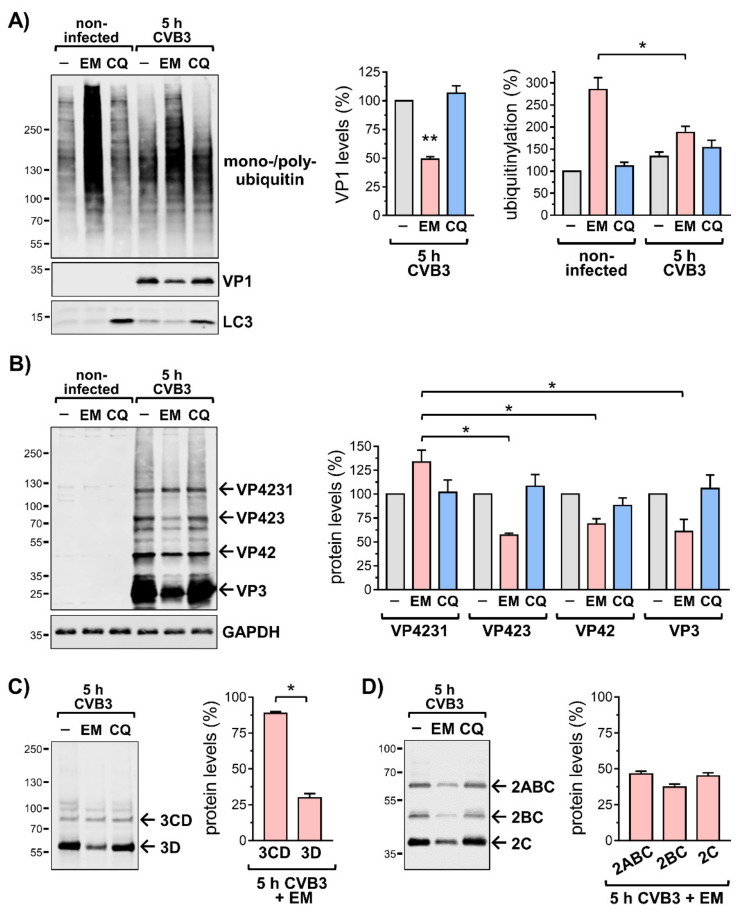
Effect of proteasome and lysosome inhibitors on CVB3 replication. (**A**–**D**) HeLa cells were treated for 5 h −/+ CVB3 (MOI 1) −/+ 200 nM epoxomicin (EM) or 100 µM chloroquine (CQ). (**A**) Total cell lysates were analyzed by immunoblotting of mono-/poly-ubiquitinylation, viral capsid protein VP1, and autophagosome marker LC3 (the lipidated LC3-II signal was enhanced, the higher molecular weight LC3-I form yielded weak signal intensity). The bar charts summarize the densitometric analysis of anti-ubiquitin and anti-VP1 immunosignals (untreated = 100%, *n* = 3), * *p* < 0.05, ** *p* < 0.05. (**B**) Immunoblotting using an antiserum raised against the fragment VP423 of the CVB3 polyprotein. The arrows indicate immunosignals of precursor proteins and cleavage products corresponding to VP4231 (~94 kDa), VP423 (~62 kDa), VP42 (~36 kDa), and VP3 (~26 kDa). GAPDH was immunoblotted as loading control. The bar chart summarizes the normalized levels of indicated immune signals in CVB3 infected cells treated with EM (respective signal in untreated = 100%, *n* = 3), * *p* < 0.05. (**C**) Immunoblotting of CVB3 protein 3D and its precursor 3CD using a polyclonal antiserum raised against protein 3D. The bar chart summarizes the densitometric analysis of anti-3CD and anti-3D immunosignals (respective signal in untreated = 100%, *n* = 4), * *p* < 0.05. (**D**) Immunoblotting using a polyclonal antiserum raised against protein 2C. The arrows indicate immunosignals of precursor proteins and cleavage products corresponding to 2ABC (~65 kDa), 2BC (~44 kDa), and 2C (~37 kDa). The bar chart summarizes the densitometric analysis of anti-2ABC, anti-2BC and anti-2C immunosignals (respective signal in untreated = 100%, *n* = 3).

To examine the role of the UPS during the first replication cycle of CVB3 in more detail, HeLa cells were infected for five hours with CVB3 in the presence of the proteasome inhibitor epoxomicin. Analysis of total lysates by immunoblotting of the viral capsid protein VP1 demonstrated that an inhibition of proteasome-mediated degradation profoundly reduced viral replication (Figure 4A), being in line with previous reports [7,8]. Cells were also treated with the lysomotropic agent chloroquine, which inhibits degradation of proteins and organelles by autophagy, a pathway that is exploited by CVB3 to promote its replication [23]. However, chloroquine had no profound effect on the levels of VP1 (Figure 4A). The enhanced detection of lipidated LC3 (LC3-II), a marker for autophagosome formation, confirmed the efficacy of the chloroquine in both, non-infected and CVB3 treated cells.

The analysis of total lysates by immunoblotting in non-infected cells that were treated with the proteasome inhibitor epoxomicin for five hours showed a profound increase of ubiquitin conjugates that are prone to proteasomal degradation (Figure 4A and Figure S3A). However, the epoxomicin-dependent increase of protein ubiquitinylation, which involved K48-linked polyubiquitinylation (Figure S3A) was markedly reduced in CVB3 infected cells, when compared to non-infected cells. The difference of the epoxomicin-dependent increase in ubiquitin conjugates during the investigated period between non-infected and CVB3 infected cells most likely constitutes the fraction of proteins that could not achieve proper folding after synthesis and were, hence, co-translationally ubiquitinylated [24]. In CVB3-infected cells, the translation of endogenous proteins undergoes a shut-off, primarily due to the cleavage of the eukaryotic translation initiation factor 4G (eIF4G) by the viral protease 2A [25]. Therefore, less endogenous proteins are synthesized and become co-translationally ubiquitinylated. The cleavage of eIF4G occurred in CVB3 infected cells with and without epoxomicin with similar efficacy and was virtually complete under both conditions despite the reduced viral replication during proteasome inhibition (Figure S3B). A similar effect with regard to the levels of ubiquitin conjugates was achieved by treating cells with epoxomicin in presence of cycloheximide, which blocks protein translation (Figure S3C).

In contrast to CVB3 infection, the ectopic expression of Halo-tagged protein 2B or protein 3A had no effect on the inhibition of proteasome-mediated degradation and the epoxomicin-dependent accumulation of ubiquitin conjugates showed no differences to mock transfected cells (Figure S3D,E). The remaining epoxomicin-dependent increase of ubiquitin-conjugates in CVB3 infected cells would therefore represent ubiquitinylated viral proteins that add to the ubiquitinylation of membrane-bound proteins due to the membrane alterations caused by the CVB3 proteins 2B and 3A. However, these effects of a viral infection or the viral proteins 2B and 3A, respectively, on protein ubiquitinylation would not explain the impairment of viral replication on account of an inhibition of the proteasome with epoxomicin.

### 3.5. Inhibition of the Proteasome Affects CVB3 Polyprotein Processing

The immunoblotting of the viral polyprotein fragment VP4231, also designated precursor P1 that contains all viral capsid proteins, revealed that an inhibition of proteasome-mediated degradation reduced profoundly the levels of the smaller intermediates and cleavage products VP423, VP42, and VP3, as described for VP1 (Figure 4B, Figure S1A). However, the amount of the precursor VP4231 showed no epoxomicin-dependent decrease but was detected at higher levels compared to untreated or chloroquine-treated cells. This observation was corroborated by immunoblotting the molecular mass region corresponding to VP4231 using an anti-VP1 antibody, which detected the precursor in epoxomicin-treated samples also at higher intensity compared to samples of untreated or chloroquine-treated cells (Figure S4A). Furthermore, on immunoblots of the viral capsid proteins, the smear-like pattern, which suggests poly-ubiquitinylation of these proteins, was much less pronounced in samples of CVB3-infected cells that were treated with epoxomicin (Figure 4B). Considering that in epoxomicin-treated cells the precursor VP4231 is more abundant but the cleavage products show reduced levels, it can be assumed that the latter are targeted for ubiquitinylation.

The discriminative effect of proteasome inhibition was also observed with regard to the fragment 3CD, which contains the viral RNA-dependent RNA polymerase (3D) and the viral protease 3C (Figure S1A). Whereas in epoxomicin-treated cells the levels of the precursor 3CD remained stable and were similar to untreated or chloroquine-treated cells, the amount of the cleavage product 3D was profoundly reduced during proteasome inhibition (Figure 4C). The polyprotein fragment 3ABCD (precursor P3) was not properly detected and was, therefore, not included in the analysis.

In addition, we analyzed the processing of the polyprotein fragment 2ABC (P2), which contains the viral protease 2A as well as the transmembrane proteins 2B and 2C, by immunoblotting of protein 2C. However, the cleavage of P2 was not impaired by the treatment of CVB3-infected cells with epoxomicin and the levels of the precursor and its cleavage products 2BC and 2C, respectively, were reduced to a similar extent (Figure 4D). On the one hand, this finding shows that the proteasome is not involved in the processing of the CVB3 polyprotein in general but that it affects the further cleavage of particular fragments. On the other hand, the successful cleavage of the precursor P2 argues against the possibility that the applied proteasome inhibitor epoxomicin exhibits an off-target effect and affects the activities of the viral proteases (Figure S4B).

In conclusion, the impairment of viral replication on account of an inhibition of the proteasome with epoxomicin can be attributed to the disturbed processing of the CVB3 polyprotein.

## 4. Discussion

In this study, we demonstrated that an infection with CVB3 entails a locally restricted alteration of the UPS-dependent protein homeostasis, and that the activity of the proteasome is exploited for the processing of the viral polyprotein fragments P1 and P3 to ensure proper viral replication. Previous reports have described an accumulation of polyubiquitinylated proteins in CVB3-infected HeLa cells as well as in mouse hearts [7,8,26]. It was suggested that an impaired proteasomal degradation may play a role in viral pathogenesis, although the activity of the proteasome is not affected during CVB3 infection. As demonstrated in this study, the accumulation of ubiquitin conjugates is not a general feature of CVB3-infected cells, but rather locally restricted to virus-utilized membranes where viral replication and virion assembly occurs. A certain portion of these ubiquitin conjugates most likely constitutes viral proteins that could not achieve proper folding after synthesis and were, thus, co-translationally ubiquitinylated for proteasomal degradation [24]. Nevertheless, there are also reports showing that the UPS targets enteroviral proteins, including the RNA-dependent RNA polymerase of CVB3 and EV71, the protease 3C of EV71, and the membrane protein 2BC of CVB3, in order to hinder viral replication [8,27,28,29].

Although during viral infection the cellular physiology is severely impaired, no additional oxidative damage of proteins occurs at the site of CVB3 replication, which otherwise could have contributed to the local increase in protein ubiquitinylation [20]. This is rather surprising, considering the impact inflicted by viral proteases as well as viroporins on the host proteome, membrane integrity, and ion homeostasis. The virus even creates conditions that result in a reduction of oxidative damage due to the binding of redox-active glutathione by viral capsid proteins for the proper assembly of viral particles [21,22]. An increase in oxidative stress accompanying a CVB3 infection was suggested to be most likely secondary to virus-induced apoptosis [30].

As demonstrated in this study, the increase in ubiquitin conjugates occurs also independently of a viral infection during the ectopic expression of the CVB3 proteins 2B and 3A, respectively. Both protein 2B and protein 3A are transmembrane proteins that affect severely the integrity of cellular membranes and rearrange membranes for the formation of ROs [3,31]. These effects of the proteins 2B and 3A also affect the localization of endogenous membrane proteins [32,33,34]. It is conceivable that the changes in membrane architecture and protein composition are detected, and that the increased ubiquitinylation of associated proteins serves no longer as a signal for proteasomal degradation but diverts the tagged cargo via the autophagic pathway to the lysosome [35,36]. However, in CVB3-infected cells the lysosomal degradation is prevented by the viral proteases 2A and 3C, which cleave the autophagy receptor p62 as well as SNARE protein SNAP29 and adaptor protein PLEKHM1 required for autophagosome fusion [37,38].

An intriguing aspect of this study is the effect of the proteasome inhibitor epoxomicin on viral replication. The seeming contradiction that a CVB3 infection results in an accumulation of ubiquitin conjugates, but that an inhibition of the proteasome-mediated degradation of ubiquitinylated proteins impairs viral replication, is solved when considering/allowing for the pro-viral role of the proteasome with regard to the processing of the CVB3 polyprotein. The requirement for a proper ratio of viral proteins to ensure optimal replication and that too much of certain viral non-structural proteins can be disadvantageous was suggested previously [39]. It was shown for numerous members of the picornavirus family that a proper balance regarding the expression levels of the viral protease 3C as well as the viral RNA-dependent RNA polymerase 3D, is required/necessary to prevent premature cell death and ensure efficient viral replication. Both viral proteins have also been reported to be subjected to UPS-dependent proteolysis. The effect of epoxomicin on the intermediate fragments P1 and P3 and their cleavage products supports the notion that the activity of the proteasome is utilized to eliminate excess viral protein, which would otherwise interfere with protein-protein interactions or enzymatic activities. The remodeling of cellular membranes by CVB3 proteins 2B and 3A together with the entailed change in protein composition would provide a mechanism to manipulate the rate or efficiency of proteasome-mediated degradation or processing of viral proteins.

The detrimental effect of proteasome inhibition was shown in diverse cell culture models for numerous virus species, including adenovirus, human cytomegalovirus, and human immuno-deficiency virus [40]. Hence, investigating the molecular effects of proteasome inhibitors with focus on the processing of viral proteins might be a promising approach to elucidate the role of the UPS in various viral infections.

## Supplementary Materials

The following are available online at https://www.mdpi.com/article/10.3390/v13071360/s1, Figure S1: accumulation of ubiquitin conjugates at CVB3-utilized compartments, Figure S2: CVB3 protein 3A increases permeability of cellular membranes for Ca^2+^ ions, Figure S3: effect of proteasome and lysosome inhibitors on CVB3 replication, Figure S4: effect of proteasome and lysosome inhibitors on CVB3 polyprotein processing.
