# Supplementary material for: Coxsackievirus B3 Exploits the Ubiquitin-Proteasome System to Facilitate Viral Replication
Source: Viruses. 2021 Jul 13;13(7):1360. doi: 10.3390/v13071360 (PMC8310229; doi:10.3390/v13071360)
Supplement: Supplementary file 1 [file viruses-13-01360-s001.zip › viruses-1291021-supplementary.pdf]

Figure S1:

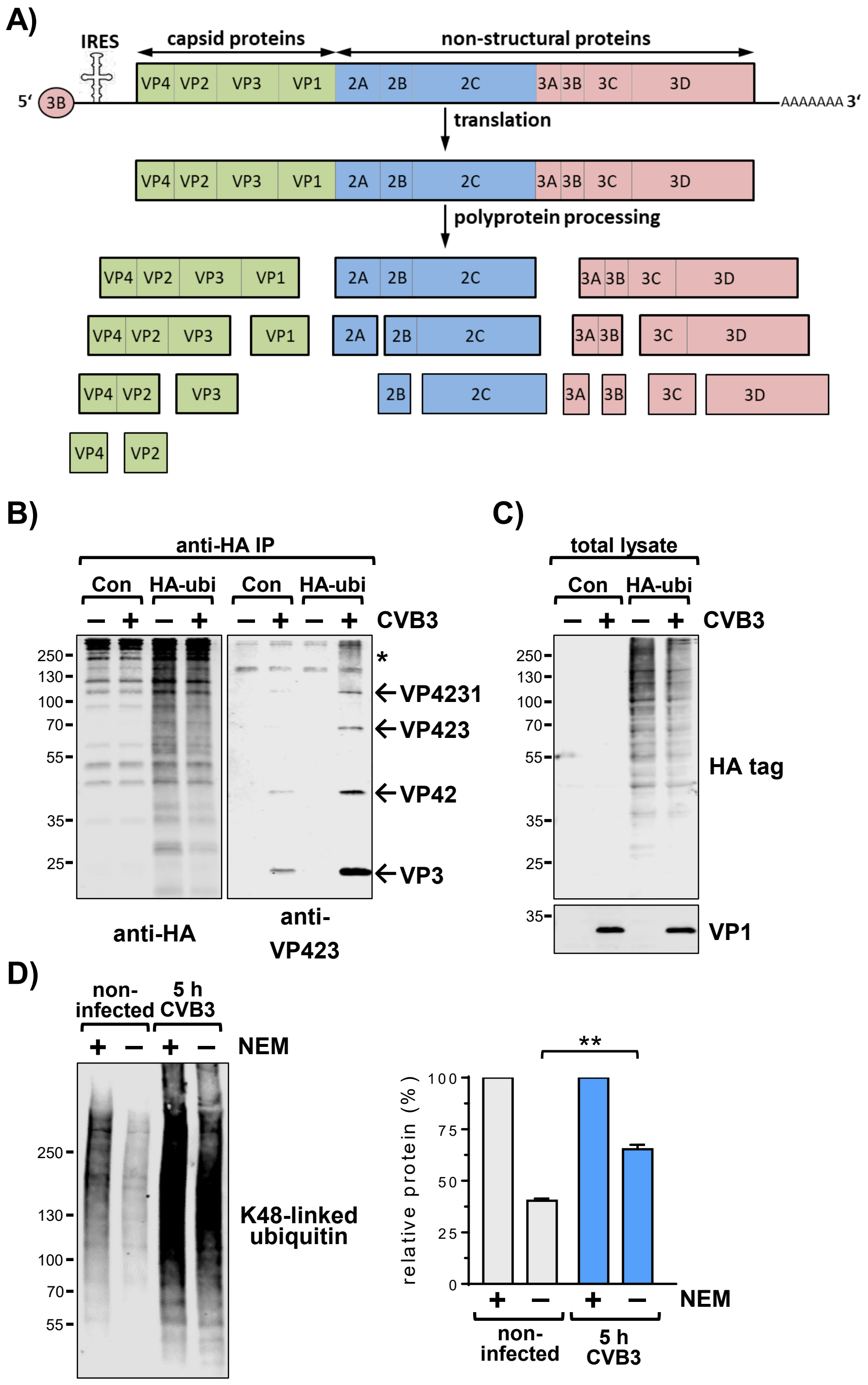

Figure S1: Accumulation of ubiquitin conjugates at CVB3-utilized compartments. **(A)** Schematic depicting the viral protease-mediated processing of the CVB3 polyprotein after translation of the viral mRNA. **(B)** Anti-HA immunoprecipitation from lysates of HA-ubiquitin-EGFP transfected (HA-ubi) HeLa cells treated for 5 h −/+ CVB3 (MOI 1) and solubilized in CHAPS supplemented lysis buffer. EGFP transfected cells were used as a control (Con). Pull-down samples were analyzed by immunoblotting of HA tag and CVB3 protein VP423. The arrows indicate immunosignals of precursor proteins and cleavage products corresponding to VP4231 (~94 kDa), VP423 (~62 kDa), VP42 (~36 kDa), and VP3 (~26 kDa). The asterisk indicates a smear in the high molecular mass region. **(C)** Total lysates of HeLa cells transfected with HA-ubiquitin-EGFP (HA-ubi) or EGFP (Con) were treated for 5 h −/+ CVB3 (MOI 1) were analyzed by immunoblotting of the HA tag and the viral capsid protein VP1. **(D)** 16k-rcf membrane fractions of HeLa cells treated for 5 h −/+ CVB3 (MOI 1) were analyzed by immunoblotting of K48-linked polyubiquitin. The bar chart summarizes the densitometric analysis of anti-ubiquitin immunosignals (+ NEM = 100%, n = 4). **p<0.01.

Figure S2:

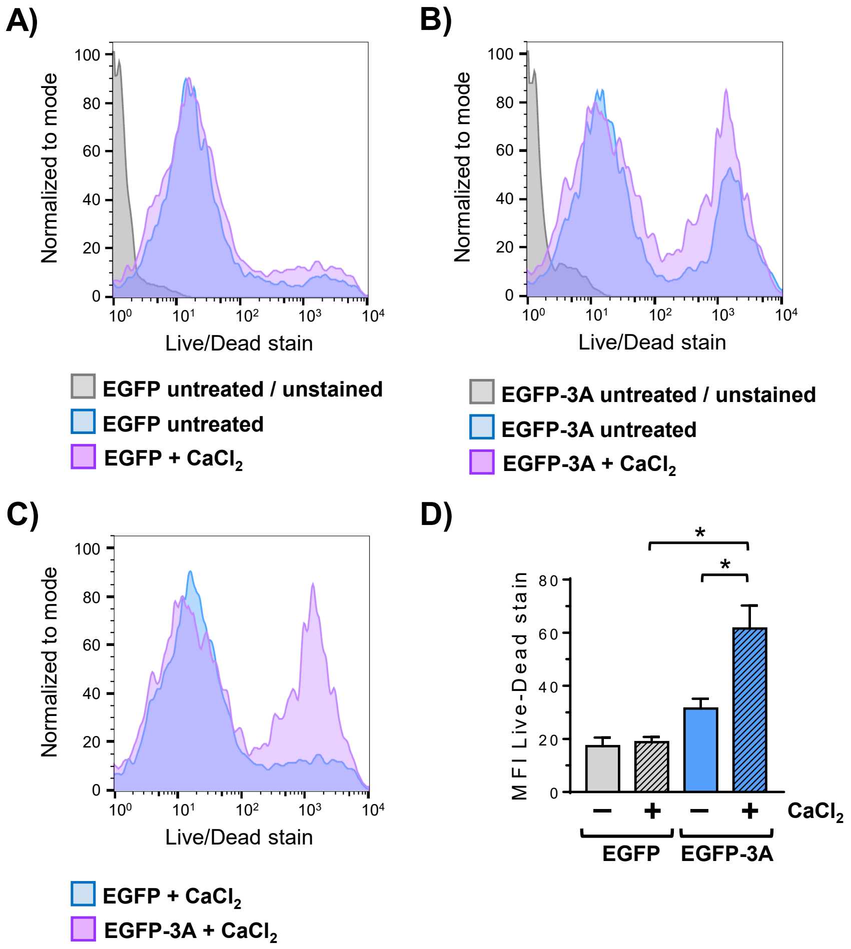

Figure S2: CVB3 protein 3A increases permeability of cellular membranes for Ca^{2+} ions. **(A–D)** Analysis of cells transfected with EGFP or EGFP-tagged CVB3 protein 3A (EGFP-3A) by flow cytometry. HeLa cells were transfected for 48 h and treated with 2.5 mM $CaCl_2$ for 60 min before collection and staining with a fixable cell viability (Live/Dead) stain. **(A)** Histograms of the Live/Dead stain in GFP-positive cells transfected with EGFP. **(B)** Histograms of the Live/Dead stain in GFP-positive cells transfected with EGFP-3A. **(C)** Comparison of the histograms of the Live/Dead stain in GFP-positive cells transfected with EGFP versus EGFP-3A and treated with $CaCl_2$. **(D)** The bar chart summarizes the mean fluorescence intensity of the Live/Dead stain in GFP-positive cells (n = 3). *$p<0.05$.

Figure S3:

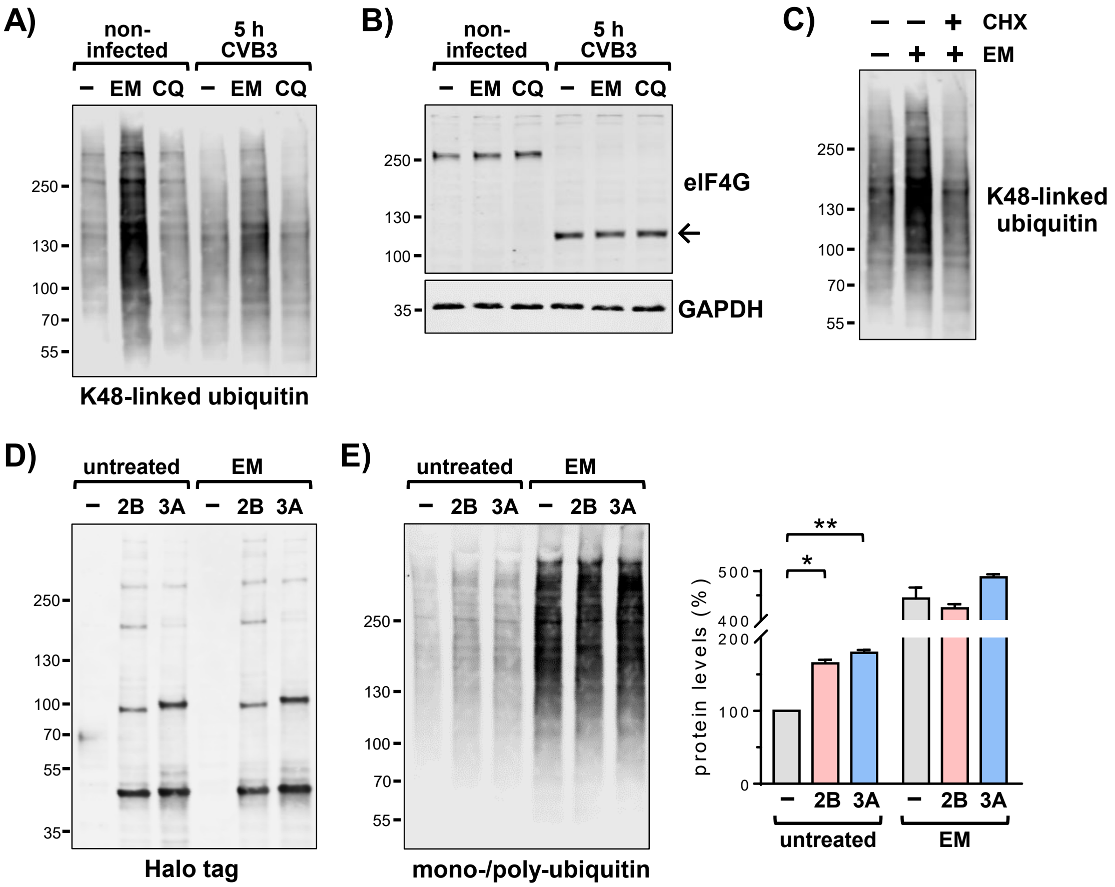

Figure S3: Effect of proteasome and lysosome inhibitors on CVB3 replication. **(A–B)** HeLa cells were treated for 5 h −/+ CVB3 (MOI 1) −/+ 200 nM epoxomicin (EM) or 100 µM chloroquine (CQ). **(A)** Total cell lysates were analyzed by immunoblotting of K48-linked polyubiquitin. **(B)** Immunoblotting of the eukaryotic translation initiation factor 4 G (eIF4G) that is cleaved by CVB3 protease 2A in infected cells. The arrow indicates the position of the N-terminal cleavage product in CVB3 infected cells. **(C)** HeLa cells were treated −/+ 200 nM epoxomicin (EM) and −/+ 50 µg/ml cycloheximide (CHX) for 5 h. Total cell lysates were analyzed by immunoblotting of K48-linked polyubiquitin. **(D–E)** HeLa cells transfected with Halo-tagged CVB3 proteins 2B or 3A and treated −/+ 100 nM epoxomicin (EM) for 14 h and processed in hypotonic buffer. The treatments started 34 h after start of transfection. Pre-cleared lysates were subjected to centrifugation at 16,000 rcf and the resulting membrane pellets were analyzed by immunoblotting of **(D)** Halo tag and **(E)** mono-/poly-ubiquitinylation. The bar chart summarizes the densitometric analysis of anti-ubiquitin immunosignals (mock = 100%, n = 3). *p<0.05, **p<0.005.

Figure S4:

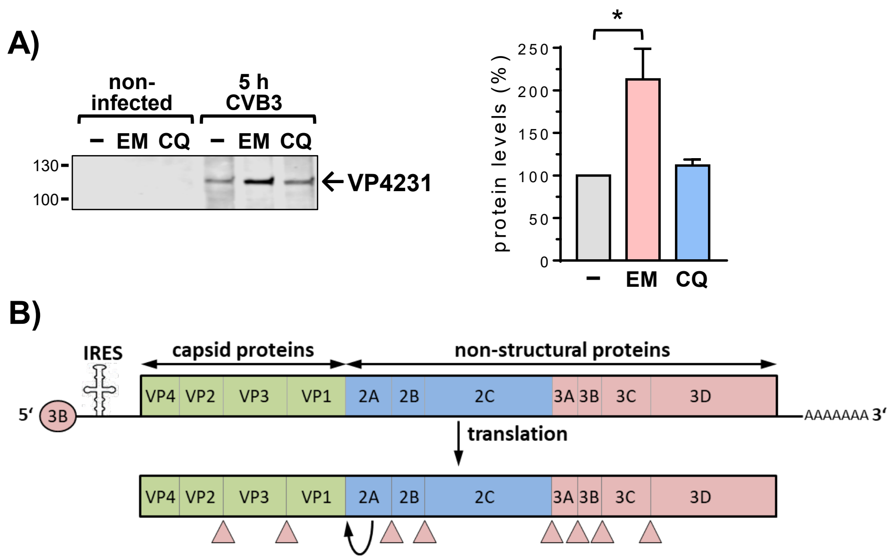

Figure S4: Effect of proteasome and lysosome inhibitors on CVB3 polyprotein processing. **(A)** HeLa cells were treated for 5 h −/+ CVB3 (MOI 1) −/+ 200 nM epoxomicin (EM) or 100 μM chloroquine (CQ). Total cell lysates were analyzed by immunoblotting of CVB3 protein VP1. The bar chart summarizes the densitometric analysis of the anti-VP1 signal corresponding to VP4231 (~94 kDa) (respective signal in untreated = 100%, n = 4). *p<0.05. **(B)** Schematic depicting the processing of the CVB3 polyprotein by the viral proteases 2A and 3C.
